# Joint Scientific Consultation Eligibility Criterion: Hubris or Naïveté

**DOI:** 10.3390/jmahp13020022

**Published:** 2025-05-15

**Authors:** Mondher Toumi, Bruno Falissard, Laurent Boyer, Pascal Auquier

**Affiliations:** 1 CEReSS/UR3279—Health Services Research and Quality of Life Center, Aix-Marseille University, 13007 Marseille, France; laurent.boyer@univ-amu.fr (L.B.); pascal.auquier@univ-amu.fr (P.A.); 2 CESP, INSERM U1018, Université Paris-Saclay, 91190 Villejuif, France; bruno.falissard@gmail.com

The eligibility criteria for Joint Scientific Consultation (JSC) raise important questions about the approach taken by the Member State Coordination Group on Health Technology Assessment (HTACG). Two main requirements must be met for eligibility: first, the product must be subject to Joint Clinical Assessment (JCA), and second, the clinical investigations must still be in the planning phase. The guidance for selecting medicinal products for JSC, updated on 21 November 2024 [1], interprets the second criterion as “*the pivotal study has not yet started and is still at a stage where the scientific recommendations of the HTACG could be taken into consideration in the final study protocol* (i.e., *the protocol has not been submitted to any regulatory authorities*)”. Health Technology Developers (HTDs) must strictly comply with this requirement to qualify for JSC. However, it appears that this critical aspect may have been overlooked or dismissed as trivial, as there has been little or no discussion regarding its importance.

This situation raises concerns about either an inflated sense of confidence or a significant lack of awareness on the part of the newly formed HTACG—an entity that is neither an official committee nor a fully operational body [2]. The HTACG has not yet begun any activities; its scope is extremely limited, its impact remains questioned and unproven, and its future will be rediscussed by the European Commission in 2028 [3]. It seems ambitious for such a group to attempt to supersede established authorities such as the European Medicines Agency (EMA) and the Food and Drug Administration (FDA), which have decades of experience in scientific consultation and initiated a parallel scientific advice process back in 2005 [4].

In terms of market context, the United States (U.S.) pharmaceutical market was valued at USD 602 billion in 2023 [5], translating to approximately USD 1800 per capita, while Europe’s market stood at USD 285 billion with USD 462 per capita [6]. The U.S. accounts for 53.3% of the global pharmaceutical market compared to Europe’s 22.7%. Furthermore, between 2018 and 2023, new medicine revenues were dominated by the U.S. at 67.1%, while Europe contributed only 15.8% [7]. Given these figures, it is unlikely that HTDs will prioritize JSC in the EU without first discussing their development plans with the FDA, and likely the EMA as well. Although parallel scientific advice is possible between EMA and the JSC subgroup [8], it remains to be seen how effectively this will function.

Despite being termed a Health Technology Assessment (HTA) regulation, the European HTA (EU-HTA) primarily addresses less critical elements of the HTA process. It reviews clinical evidence without contextual analysis and lacks critical evaluative judgment [2]. The most crucial steps—contextualization and appraisal—remain deliberative processes delegated to Member States (MSs) [2].

For instance, onasemnogene abeparvovec (ZOLGENSMA^®^) in France might not have qualified for reimbursement under a decontextualized analysis due to insufficient comparative data and small sample sizes; however, it received a Clinical Added Value (ASMR III) rating because of its significant therapeutic outcomes [9]. Similarly, while a decontextualized assessment might have excluded ZOLGENSMA^®^, the National Institute for Health and Care Excellence (NICE) endorsed it for Type I and II Spinal Muscular Atrophy (SMA) by incorporating contextual factors and patient perspectives [10]. Context is critical in reimbursement decisions.

Both the French National Health Authority (HAS) [11] and the German Federal Joint Committee (G-BA) [12] have indicated that additional analyses beyond the JCA report will be required for evaluating medicinal products submitted to their agencies. Therefore, the actual impact of JCA on national HTA and reimbursement decisions remains uncertain. In this context, it is surprising that the HTACG, yet to be established and survive, seeks to be the first to comment on clinical development plans. It would have been more appropriate to request that the final version of the protocol not be endorsed by any regulatory authority, thus leaving room for further adjustment eventually requested by the JSC group. It is likely that this mandatory eligibility condition for JSC will not be consistently enforced in practice and may require rapid revision for consistency.

## Abbreviations

The following abbreviations are used in this manuscript:

ASMR	Clinical Added Value
EMA	European Medicines Agency
EU-HTA	European Health Technology Assessment
FDA	Food and Drug Administration
G-BA	German Federal Joint Committee
HAS	French National Health Authority
HTA	Health Technology Assessment
HTACG	Member State Coordination Group on Health Technology Assessment
HTDs	Health Technology Developers
JCA	Joint Clinical Assessment
JSC	Joint Scientific Consultation
MSs	Member States
NICE	National Institute for Health and Care Excellence
SMA	Spinal Macular Atrophy
U.S.	United States
